# Supplementary figures and images for: Adverse events of COVID-19 vaccines in pregnant and postpartum women in Brazil: A cross-sectional study
Source: PLoS One. 2023 Jan 13;18(1):e0280284. doi: 10.1371/journal.pone.0280284 (PMC9838840; doi:10.1371/journal.pone.0280284)

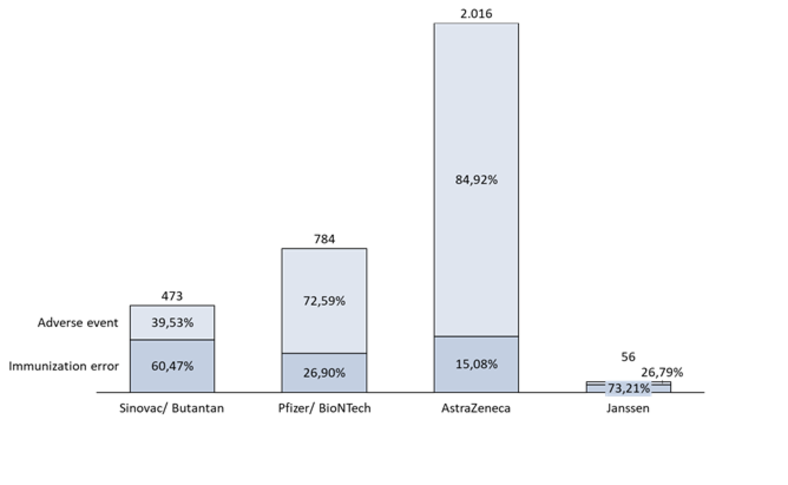

Supplement: S1 Fig — *AstraZeneca includes the vaccines ChAdOx1 nCoV-19 and BBV152. (TIF) [file pone.0280284.s001.tif]
