# Supplementary material for: Adverse events of COVID-19 vaccines in pregnant and postpartum women in Brazil: A cross-sectional study
Source: PLoS One. 2023 Jan 13;18(1):e0280284. doi: 10.1371/journal.pone.0280284 (PMC9838840; doi:10.1371/journal.pone.0280284)
Supplement: S2 Table — AE: Adverse Events; 1 AstraZeneca includes the vaccines ChAdOx1 nCoV-19 and BBV152; *AE reported as “COVID-19”, “PCR positive to COVID-19” and the like were classified as inconclusive; **AE reported as “vaccination error”, “inadvertent exposure to vaccine”, “contraindication” was classified as inconsistent. (PDF) [file pone.0280284.s003.pdf]

S3 Table. Frequency of adverse events according to the type and severity

	Pregnant										Postpartum								
	Sinovac/ Butantan		Pfizer/ BioNTech		AstraZeneca[1]		Janssen		Total		Sinovac/ Butantan		Pfizer/ BioNTech		AstraZenecai		Janssen	Total	
	AE	%	AE	%	AE	%	AE	%	AE	%	AE	%	AE	%	AE	%	AE %	AE	%
Adverse Event Type																			
Maternal	26	14.53%	47	8.39%	43	2.53%	1	6.67%	117	4.78%	0	0	0	0	0	0	- -	0	0
Local	11	6.15%	75	13.39%	204	12.04%	2	13.33%	292	11.93%	0	0	2	16.67%	1	5.56%	- -	3	7.69%
Systemic	127	70.95%	433	77.32%	1436	84.76%	12	80.0%	2008	82.03%	7	77.78%	10	83.33%	17	94.44%	- -	34	87.18%
Inconclusive*	13	7.26%	5	0.89%	9	0.53%	0	0	27	1.1%	2	22.22%	0	0	0	0	- -	2	5.13%
Inconsistent**	2	1.12%	0	0	2	0.12%	0	0	4	0.16%	0	0	0	0	0	0	- -	0	0
Severity																			
Severe	36	20.11%	96	17.14%	93	5.49%	1	6.67%	226	9.23%	4	44.44%	1	8.33%	0	0	- -	5	12.82%
Non-severe	143	79.89%	463	82.68%	1599	94.39%	14	93.33%	2219	90.65%	5	55.56%	11	91.67%	18	100.0%	- -	34	87.18%
Ignored	0	0	1	0.18%	2	0.12%	0	0	3	0.12%	0	0	0	0	0	0	- -	0	0

AE: Adverse Events; [1] AstraZeneca includes the vaccines ChAdOxl nCoV-19 and BBV152;

*AE reported as "COVID-19", "PCR positive to COVID-19" and the like were classified as inconclusive;

**AE reported as "vaccination error", "inadvertent exposure to vaccine", "contraindication" was classified as inconsistent.
