# Supplementary material for: Adverse events of COVID-19 vaccines in pregnant and postpartum women in Brazil: A cross-sectional study
Source: PLoS One. 2023 Jan 13;18(1):e0280284. doi: 10.1371/journal.pone.0280284 (PMC9838840; doi:10.1371/journal.pone.0280284)
Supplement: S3 Table — AE: Adverse event; 1AstraZeneca includes the vaccines ChAdOx1 nCoV-19 and BBV152. (PDF) [file pone.0280284.s004.pdf]

S4 Table. Frequency of most common adverse events experienced by pregnant and postpartum women receiving COVID-19 vaccines and who reported an AEFI

	Sinovac/Butantan		Pfizer/BioNTech		AstraZeneca[1]		Janssen		Total	
	AE (n= 187)	%	AE (n= 572)	%	AE (n= 1712)	%	AE (n= 15)	%	AE (n=2,486)	%
Maternal										
Spontaneous abortion	9	4.81%	28	4.9%	21	1.23%	1	0.066%	59	2.37%
Pregnancy bleeding	6	3.21%	3	0.52%	11	0.64%	0	0%	20	0.80%
Neonatal death	3	1.6%	9	1.57%	1	0.06%	0	0%	13	0.52%
Premature birth	1	0.53%	3	0.52%	3	0.18%	0	0%	7	0.28%
Abdominal pregnancy	3	1.6%	0	0%	0	0%	0	0%	3	0.12%
Non-maternal										
Headache	20	10.7%	77	13.46%	360	21.03%	4	28.57%	461	18.54%
Fever	13	6.95%	46	8.04%	282	16.47%	2	14.29%	343	13.8%
Myalgia	12	6.42%	37	6.47%	206	12.03%	1	7.14%	256	10.3%
Pain	3	1.6%	35	6.12%	149	8.7%	2	14.29%	189	7.6%
Vomit	2	1.07%	20	3.5%	67	3.91%	2	14.29%	91	3.66%

Chills	4	2.14%	8	1.4%	77	4.5%	0	0%	89	3.58%
Nausea	2	1.07%	19	3.32%	62	3.62%	1	7.14%	84	3.38%
Abdominal pain	9	4.81%	22	3.85%	44	2.57%	1	7.14%	76	3.06%
Diarrhea	3	1.6%	20	3.5%	40	2.34%	0	0%	63	2.53%
Arm Pain	0	0%	26	4.55%	34	1.99%	0	0%	60	2.41%
Asthenia	6	3.21%	15	2.62%	35	2.04%	0	0%	56	2.25%
Fatigue	2	1.07%	14	2.45%	39	2.28%	0	0%	55	2.21%
Cough	7	3.74%	28	4.9%	17	0.99%	0	0%	52	2.09%
Dyspnea	5	2.67%	15	2.62%	31	1.81%	0	0%	51	2.05%
Runny nose	7	3.74%	23	4.02%	16	0.93%	0	0%	46	1.85%
Sore throat	4	2.14%	11	1.92%	22	1.29%	0	0%	37	1.49%
Edema	4	2.14%	9	1.57%	14	0.82%	0	0%	27	1.09%

AE: Adverse event; [1]AstraZeneca includes the vaccines ChAdOx1 nCoV-19 and BBV152.
